# An Unusual Case of Recurrent Gingival Epstein-Barr Virus-Positive B-Cell Lymphoproliferation

**DOI:** 10.7759/cureus.42262

**Published:** 2023-07-21

**Authors:** Pauline Le Gatt, Pauline Quilhot, Geraldine Lescaille, Juliette Rochefort

**Affiliations:** 1 Oral Surgery, Hopital de la Pitié-Salpêtrière, Paris, FRA; 2 Pathology and Laboratory Medicine, Hopital de la Pitié-Salpêtrière, Paris, FRA; 3 Oral Surgery, Hôpital de la Pitié-Salpêtrière, Paris, FRA

**Keywords:** gingival lesion, b lymphocytes, immuno suppresion, ebv-positive, lymphoproliferation

## Abstract

Epstein-Barr virus (EBV)-positive mucocutaneous ulceration commonly presents as a B-cell lymphoproliferative process with manifold aspects. There is scarce data on its oral manifestation in the scientific literature. We report the case of a 57-year-old male immunocompromised renal transplant patient who developed recurrent chronic and symptomatic mucosal ulceration facing the mandibular incisor teeth. Pathological examination with microscopic and immunohistochemistry studies revealed a B plasma cell infiltration as well as positive staining for EBV, leading to a diagnosis of EBV-positive mucocutaneous ulceration with B-cell lymphoproliferation after extensive workup. Treatment with rituximab was implemented and led to the healing of the lesion.

This lesion develops in geriatric and immunodeficient patients and may require oncological therapies. While it is generally associated with an excellent prognosis, it may be indicative of underlying immunosuppression, and hence oral cavity specialists must be aware of this particular entity.

## Introduction

Lymphoproliferation refers to the polyclonal proliferation of B or T lymphocytes in different lymphoid tissue territories. The primary cutaneous lymphoproliferative syndrome is described in the updated World Health Organization-European Organization for Research and Treatment of Cancer (WHO-EORTC) 2018 classification, including cutaneous T-cell and B-cell lymphomas [1]. Epstein-Barr virus (EBV)-positive mucocutaneous ulceration has been included in the latter category and is characterized by the rapid and unique development of single ulceration in patients with drug or age-related immune deficiency involving the skin, oral mucosa, or gastrointestinal mucosa [2].

There are very few case reports involving an oral location of EBV-positive ulceration. However, knowledge and early diagnosis of this entity could enable prompt identification of major immune deficiencies. In this report, we present a case of a male patient with B-cell lymphoproliferation.

## Case presentation

The patient was a 57-year-old man who consulted with the odontology emergency department for the first time in October 2021 for persistent gingival ulceration, which had lasted for one month, was symptomatic, and unresolved after six days of therapy with amoxicillin 2 grams per day as prescribed by his attending physician. The patient had a medical history of familial Mediterranean fever resulting in digestive and renal AA amyloidosis treated by renal transplantation in 2006 and associated with the use of long-term immunosuppressants (prednisolone, tacrolimus, and mycophenolic acid). He had also suffered from coronary syndrome, which led to stent placement and intake of DL-lysine acetylsalicylate, as well as hypothyroidism stabilized by levothyroxine.

**Figure 1:**
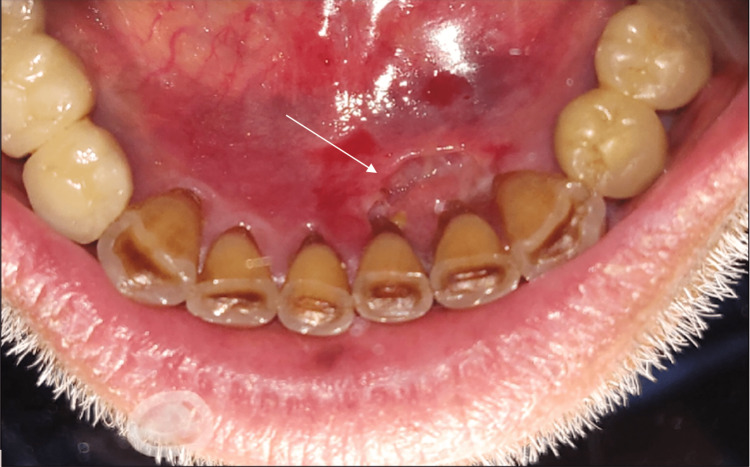
Endobuccal photograph of lingual ulceration of teeth 41, 31, and 32

Clinical examination revealed a well-demarcated gingival ulceration of 1 cm in diameter, budding with a necrotic background with hemmed edges initially lingual to the mandibular incisors (Figure 1).

**Figure 2:**
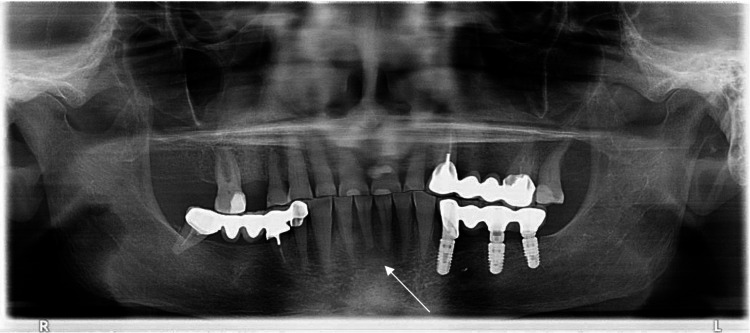
Panoramic radiograph during the emergency consultation showing no bone involvement

A panoramic radiograph was performed and showed no bone involvement in front of the lesion (Figure 2).

**Figure 3:**
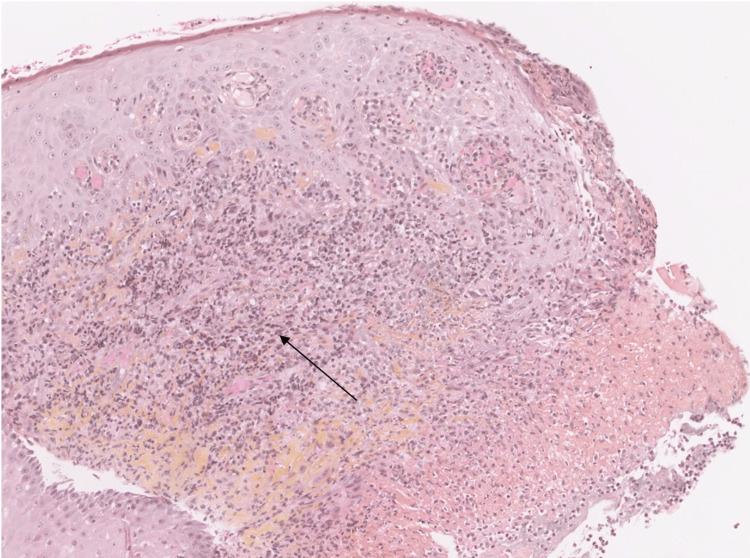
Pathological section with hematoxylin-eosin saffron staining showing the large mononuclear lymphoid cells infiltration within the chorion The arrow points to a lymphoid cell

In light of this clinical presentation, we put forward the diagnostic hypotheses of squamous cell carcinoma, an infectious ulceration - more specifically tubercular, syphilitic, or EBV-related - or an ulceration caused by trauma. Pathological examination with microscopic analysis revealed a site of a predominantly mononuclear large inflammatory infiltrate, with patches of medium to large lymphoid cells throughout the connective tissue (Figure 3).

**Figure 4:**
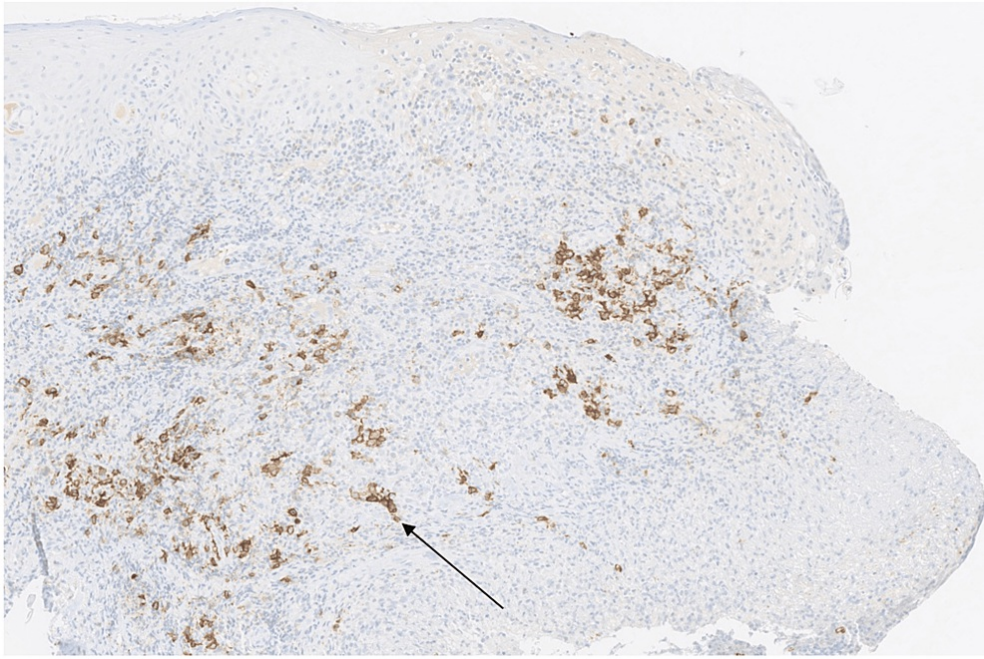
Anatomopathological section of lymphoid cells with immunohistochemical marking of the CD20 molecule The arrow points to a CD20-positive lymphoid cell

Immunohistochemistry revealed positive labeling of the large lymphoid cells for CD20 and CD30, which are specific markers of mature B cells, indicating that the infiltrate is composed of B plasma cells (Figure 4).

**Figure 5:**
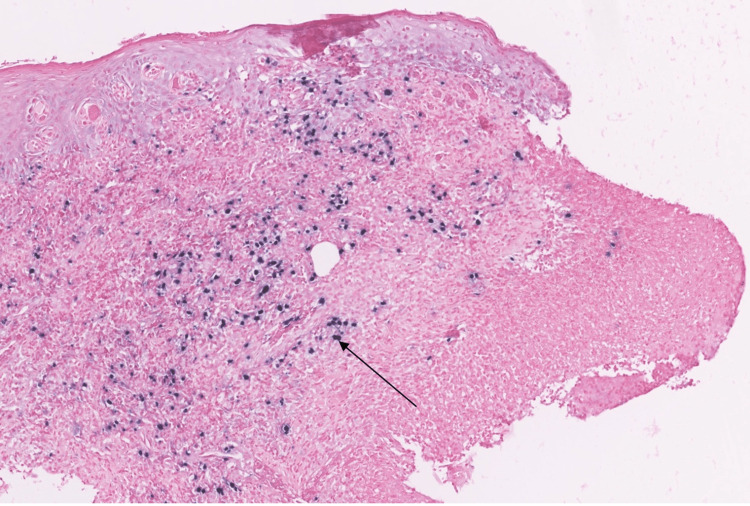
Pathological section of lymphoid cells infiltration with EBV labeling with EBER probe The arrow points to an EBV-positive lymphoid cell EBV: Epstein-Barr virus; EBER: EBV-encoded RNA

These same cells were positive for the EBV marker based on the EBV-encoded RNA (EBER) probe (Figure 5).

**Figure 6:**
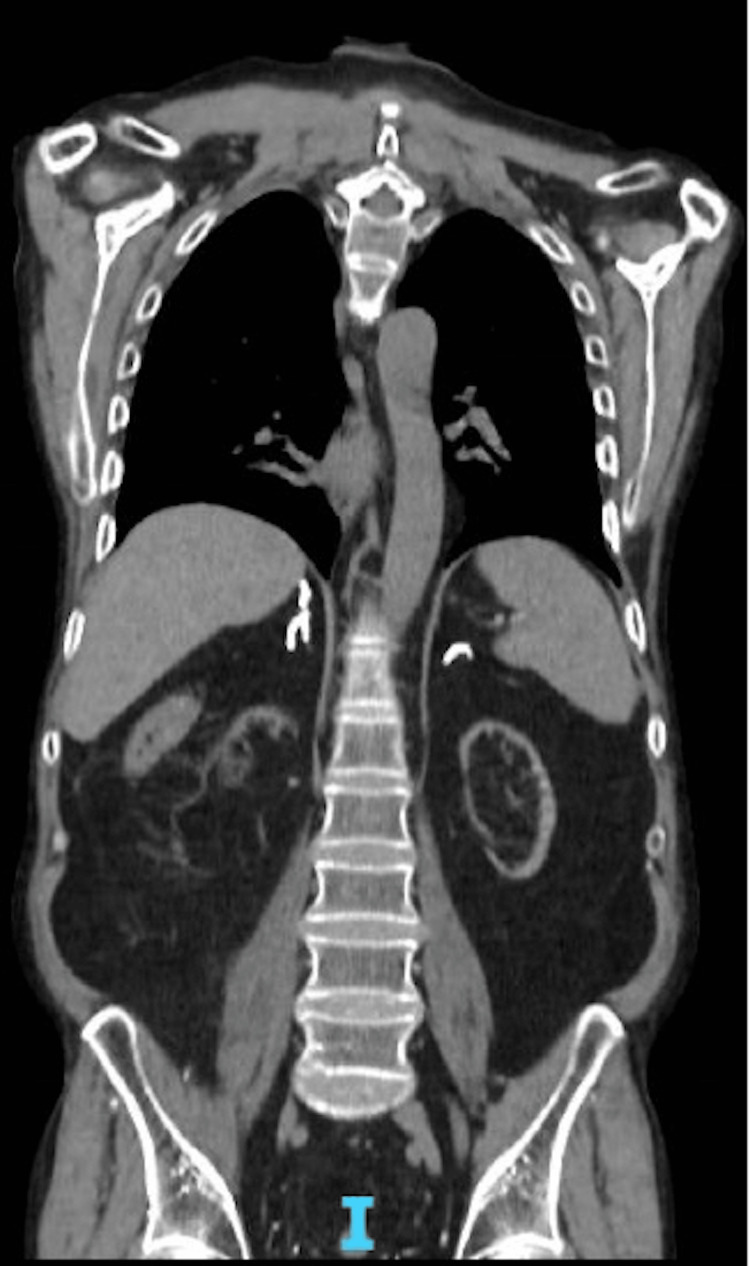
PET scan of the thoraco-abdominopelvic section with no hypermetabolic focus PET: positron emission tomography

The clinical analysis and histological findings thus revealed mucosal ulceration with CD20+, EBV+ B-cell lymphoproliferation. Given this diagnosis, an extensive workup including a blood count, EBV viral load, and a positron emission tomography (PET) scan was performed. The PET scan did not reveal any hypermetabolic focus outside the local focus (Figure 6).

The EBV viral load was positive, and the blood count showed no particularities compared to those performed previously during his follow-ups (Table 1).

**Table 1:** Blood counts

	October 21	February 20	Reference range
Leukocytes (G/L)	5.85	7.13	4.00-10.00
Erythrocytes (T/L)	4.53	4.52	4.50-10.50
Hemoglobin (g/100 ml)	12.7	12.8	13.0-17.0
Hematocrit (%)	40.2	42.2	40.0-50.0
Mean corpuscular volume (u^3^)	89	93	82.0-98.0
Mean corpuscular hemoglobin concentration (g/100 mg)	31.6	30.3	32.0-35.0
Mean corpuscular hemoglobin (pg)	28.0	28.3	27,0-33,0
Neutrophils (/mm^3^)	4580	5476	1500-7000
Eosinophils (/mm^3^)	0	21	<400
Basophils (/mm^3^)	30	21	<100
Lymphocytes (/mm^3^)	760	941	1500-4000
Monocytes (/mm^3^)	480	670	200-800
Platelets (G/L)	156	148	150-400
Creatinine (mg/L)	18.8	19.8	7.3-11.8
Glomerular filtration rate (mL/min/1.73 m^2^)	33	36.93	>90
C-reactive protein (mg/L)	4.69	3.2	<5.0
Aspartate aminotransferase (U/L)	39	32	5-34
Alanine transaminase (U/L)	28	21	<55
Gamma-glutamyl transferase (U/L)	23	27	12-64
Alkaline phosphatase (U/L)	185	214	40-120

In light of these examination results, the final diagnosis of EBV-positive mucocutaneous ulceration complicated by immunosuppressive therapy was established according to the WHO 2016 classification of lymphomas. The main differential diagnosis was EBV-positive polymorphic post-transplant lymphoproliferation characterized by the clinical presentation of a tissue mass. It was ruled out because the patient presented clinical signs of ulceration and his lesion developed several years after transplantation.

**Figure 7:**
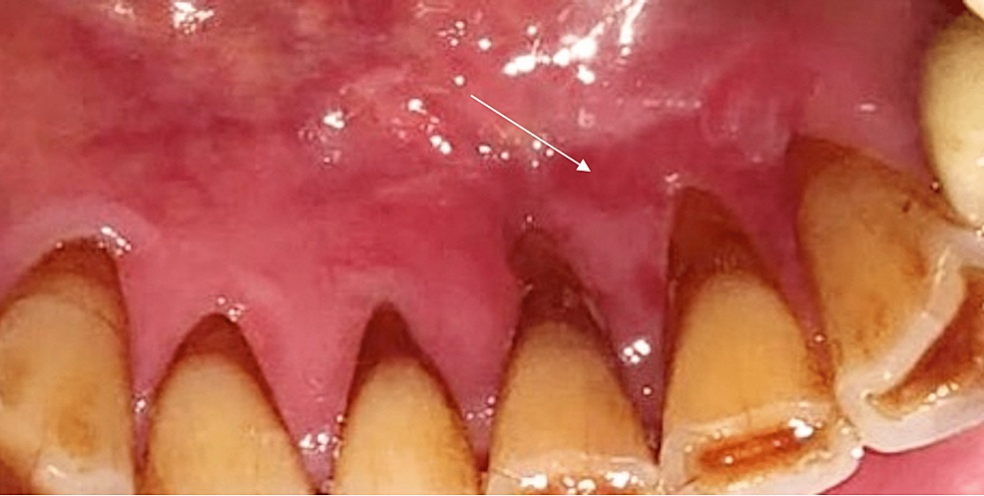
Lingual gingiva view of the lingual ulceration after the first treatment

Treatment then consisted of the initiation of rituximab in November 2021, given that reducing immunosuppressants in this patient was not feasible due to the high risk of graft rejection. At the follow-up one week after the first intake of rituximab, an improvement in the lesion was observed (Figure 7).

**Figure 8:**
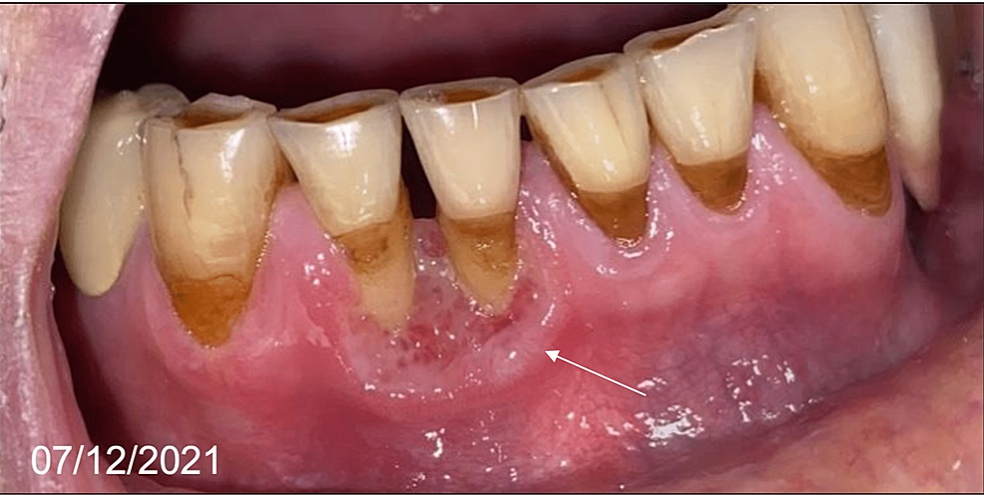
Labial gingiva view of the vestibular recurrence in December 2021

However, a recurrence in the vestibular gingiva was observed one month after healing was achieved (Figure 8), which was confirmed by a new biopsy.

**Figure 9:**
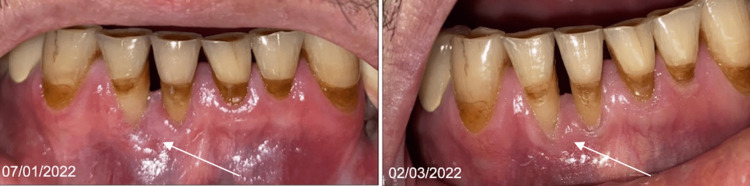
Labial gingiva views of improvement in January and March

A new course of rituximab was then administered involving one injection every 21 days, which again resulted in improvement at one month and stabilization of healing with four courses. No recurrence was observed at follow-up visits until April 2022 (Figure 9).

**Figure 10:**
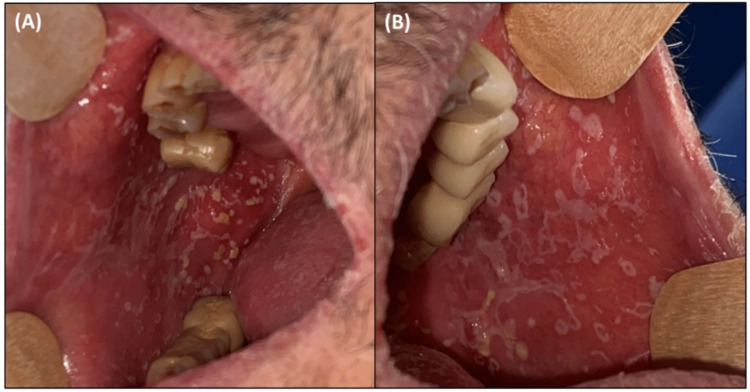
Intraoral photographs of oral candidiasis

The patient subsequently developed multiple infections, including pulmonary by severe acute respiratory syndrome coronavirus 2 (SARS-CoV-2), colon by Clostridium difficile, and mucocutaneous by Candida albicans (Figure 10). His general condition rapidly deteriorated, ultimately leading to his death in August 2022.

## Discussion

There is a lack of consensus in the literature regarding the management of mucosal ulceration involving EBV-positive lymphoproliferation. Several treatment modalities have been described, alone or in combination, depending on the local prognosis of the lesion: reduction or substitution of immunosuppressants, rituximab, chemotherapy, or radiotherapy [3]. In our patient, treatment with rituximab was undertaken due to the impossibility of reducing immunosuppressive drug dosage because of the high risk of rejection of the renal graft as well as the patient's use of a semi-automatic defibrillator.

This entity is more commonly observed in the mouth, especially in the tonsils, tongue, buccal mucosa, and palate (52%) [4], than on the skin (29%) or in the gastrointestinal tract (19%) [5] because the oral cavity constitutes the primary site of viral infection through saliva contamination. However, in the literature, it is often associated with inflammatory conditions such as rheumatoid arthritis [6] or Crohn's disease [7].

The main differential diagnosis to be considered for this entity remains the EBV-related diffuse large B-cell lymphoma of the elderly, which has a much poorer prognosis [8]. Also, according to some researchers, local trauma could lead to the activation of a chronic inflammatory reaction leading to ulceration and proliferation of virus-infected B cells [9]. The prognosis of mucosal ulceration with EBV-positive B-cell lymphoproliferation is mostly excellent with appropriate treatment of the patient [8]. Patients with this condition are usually compromised by extensive immunosuppressive therapy, and treatment of this condition often adds to the burden of care. However, there is still a risk of progression into lymphoma [10].

More than 90% of the population is infected with EBV [11]. The virus infects mainly B lymphocytes and remains latent in memory B cells [12]. In immunocompromised patients, the virus can proliferate more easily and lead to more severe pathologies, such as Burkitt lymphoma or oral scalp leukoplakia [11]. Thus, EBV-positive infection can be indicative of immunosuppression of various etiologies. In our case, where the patient was under immunosuppressive treatment, it was difficult to understand the reasons for EBV-positive reactivation since the immunosuppressive treatments had been in place since 2006, and the white blood cell levels did not show any notable collapse compared to the levels previously documented. However, this oral EBV-positive infection was the first in a series of multiple infections that eventually led to the patient's death. Thus, the clinical manifestation of EBV positivity by an oral lesion is certainly indicative of general deterioration.

The knowledge and early diagnosis of this entity could enable the prompt identification of major immune deficiencies because only highly immunocompromised patients are affected [6]. A multidisciplinary follow-up involving hematology and oral surgery is then necessary to avoid any recurrence or malignant transformation of the pathology.

## Conclusions

While EBV-positive mucosal ulceration is a rare entity with an excellent prognosis, it may be indicative of severe immunosuppression and impaired general condition. Extensive and rigorous follow-up of patients with this condition is essential given the risk of developing lymphoma as well as their immunosuppressed status.
